# A comparison between ARIA and visual analogic scale methods for classifying allergic rhinitis severity

**DOI:** 10.1186/1939-4551-8-S1-A5

**Published:** 2015-04-08

**Authors:** Eduardo Costafsilva, Tatiana Menezes Monteiro, Anna Carolina Nogueira Arraes, Gabriela Andrade C Dias, Denise Lacerda Pedrazzi, Guilherme Loureiro Werneck, Maria Ines Perelló

**Affiliations:** 1 State University of Rio De Janeiro, Brazil; 2 Federal University of Rio De Janeiro, Brazil

## Background

There are different classification systems for allergic rhinitis (AR) severity, whose are used to guide treatment. Clinical observation suggests that ARIA method, made by doctors, and visual analogic scale of symptoms (VASS), made by patients, do not obtain the same results in many occasions. The aim of this study was to compare the results of these methods applied at the same time in a cohort of adolescents and adults with AR under specialized outpatient care.

## Methods

Retrospective study of clinical records of patients with AR in treatment between March-2011 and August-2012 at an university hospital in Rio de Janeiro/Brazil, where both classifications have been used routinely since 2010. Four hundred clinical sheets were reviewed, we excluded patients under 12 years old, without at least one cutaneos test positive to aeroallergens and those with incorrect clinical data. Kappa coefficient (Stata 11) was used to measure agreement between them using 2 (mild and moderate/severe), 3 (mild, moderate and severe) and 6 categories (also considering intermittent and persistent grades), according to original and modified ARIA classifications.

## Results

We included clinical records of 124 patients: 88 were women (71%), the median age was 39 years (perc25-75=17-55) and 77 (62%) had associated asthma. Using ARIA modificated method (ARIAm - Valero at al. 2007), they were classified as mild=55 (44.3%), moderate=56 (45.1%) and severe=13 (10.4%). Using VASS, patients classified themselves as mild=35 (28.2%), moderate=68 (54.8%) and severe=21 (16.9%). Kappa analysis in the entire sample showed low agreement at 2, 3 or 6 comparison levels between the classifications (k=0.30, 0.39 and 0.37, respectively). The classifications were different in 51 (41.1%) patients when we compared 3 level of severity with lower agreements at 3 or 6 levels of comparison in females, in patients older than 18 years and in those with associated asthma. Better concordance was achieved in patients younger than 18 years (substantial), in patients without asthma (moderate) and in males (moderate).

## Conclusions

Our findings suggest that many patients have a different perception of severity of their disease using VASS when compared with results from ARIA classification, with a low to moderate concordance between the two methods. Presence of asthma, age and gender seems to influence these results. Large studies comparing the outcomes using both methods to guide treatment may help us to define the better one to make therapeutic decisions in clinical practice.

